# 2-(Carb­oxy­meth­yl)imidazo[1,2-*a*]pyridin-1-ium chloride

**DOI:** 10.1107/S1600536812051549

**Published:** 2013-01-09

**Authors:** Wen-Yu Yin

**Affiliations:** a Department of Chemistry & Materials Engineering, Jiangsu Laboratory of Advanced Functional Materials, Changshu Institute of Technology, Changshu 215500, Jiangsu, People’s Republic of China

## Abstract

In the crystal structure of the title salt, C_9_H_9_N_2_O_2_^+^·Cl^−^, the cations and anions are linked into chains parallel to [021] by O—H⋯Cl and N—H⋯Cl hydrogen bonds.

## Related literature

For the diversity of structures and the applications of compounds with an imidazole moiety, see: Catalano & Etogo (2007 ▶); Feng *et al.* (2012 ▶); Keppler *et al.* (1987 ▶); Poul *et al.* (2007 ▶); Saha *et al.* (2012 ▶); Samantaray *et al.* (2007 ▶); Takagaki *et al.* (2012 ▶).
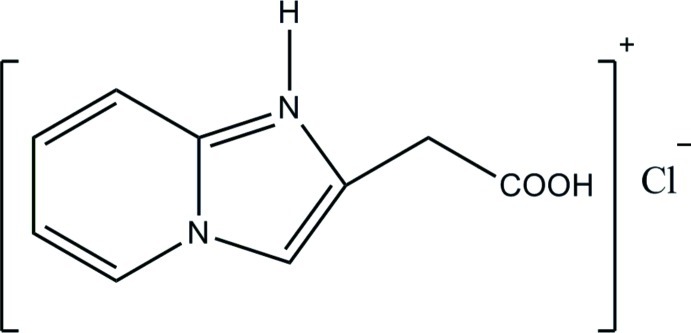


## Experimental

### 

#### Crystal data

C_9_H_9_N_2_O_2_^+^·Cl^−^
*M* *_r_* = 212.63
Monoclinic, 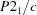

*a* = 5.4032 (8) Å
*b* = 14.722 (2) Å
*c* = 12.1055 (18) Å
β = 96.182 (4)°
*V* = 957.3 (2) Å^3^
*Z* = 4
Mo *K*α radiation
μ = 0.37 mm^−1^
*T* = 293 K
0.25 × 0.15 × 0.12 mm

#### Data collection

Rigaku Mercury diffractometer
Absorption correction: multi-scan (*CrystalClear*; Rigaku, 2005 ▶) *T*_min_ = 0.913, *T*_max_ = 0.957
7948 measured reflections
1689 independent reflections
1417 reflections with *I* > 2σ(*I*)
*R*_int_ = 0.044

#### Refinement

*R*[*F* ^2^ > 2σ(*F* ^2^)] = 0.047
*wR*(*F* ^2^) = 0.083
*S* = 1.02
1689 reflections
134 parameters
H atoms treated by a mixture of independent and constrained refinement
Δρ_max_ = 0.19 e Å^−3^
Δρ_min_ = −0.20 e Å^−3^

### 

Data collection: *CrystalClear* (Rigaku, 2005 ▶); cell refinement: *CrystalClear*; data reduction: *CrystalClear*; program(s) used to solve structure: *SHELXS97* (Sheldrick, 2008 ▶); program(s) used to refine structure: *SHELXL97* (Sheldrick, 2008 ▶); molecular graphics: *SHELXTL/PC* (Sheldrick, 2008 ▶); software used to prepare material for publication: *SHELXTL/PC* and *PLATON* (Spek, 2009 ▶).

## Supplementary Material

Crystal structure: contains datablock(s) global, I. DOI: 10.1107/S1600536812051549/hp2052sup1.cif

Structure factors: contains datablock(s) I. DOI: 10.1107/S1600536812051549/hp2052Isup2.hkl

Supplementary material file. DOI: 10.1107/S1600536812051549/hp2052Isup3.cml

Additional supplementary materials:  crystallographic information; 3D view; checkCIF report

## Figures and Tables

**Table 1:** Hydrogen-bond geometry (Å, °)

*D*—H⋯*A*	*D*—H	H⋯*A*	*D*⋯*A*	*D*—H⋯*A*
O1—H1⋯Cl1^i^	0.82	2.19	2.984 (2)	163
N2—H2*A*⋯Cl1^ii^	0.89 (3)	2.18 (3)	3.074 (2)	175 (3)

Symmetry codes: (i) 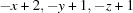; (ii) 
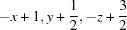
.

**Fig. 1.**
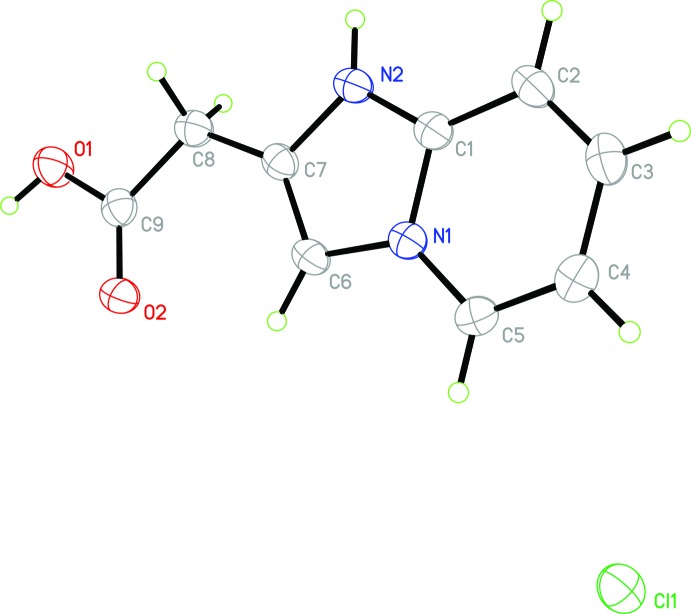
The molecular structure of the title compound, with atom labels and anisotropic displacement ellipsoids (drawn at 30% probability level).

**Fig. 2.**
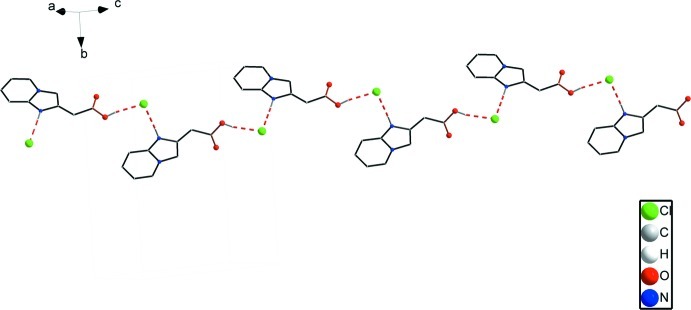
The packing of the title compound. Hydrogen bonds are shown as dashed lines. All H attached to carbon atoms were omitted for clarity.

**Fig. 3.**
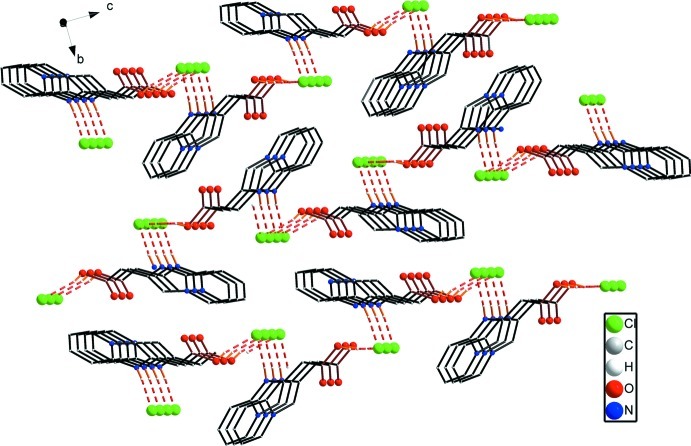
Three dimensional strucure viewed along the a axis.

## supplementary crystallographic information

### Comment

Derivatives of imidazole have received great attention for their applications in the field of biology (Catalano *et al.*, 2007; Poul *et al.*, 2007; Takagaki *et al.*, 2012;). The most pervasive is the amino acid histidine, which has an imidazole side-chain (Feng *et al.*, 2012; Samantaray *et al.*, 2007;). In recent years, many derivatives have been used as antifungal agents and bone resorption inhibitors (Keppler *et al.*, 1987; Saha *et al.*, 2012;). As illustrated in Fig. 1, the title compound is composed of one imidazo[1,2-*a*]pyridin-2-acetic acid cation and a Cl^-^ anion. The acetic acid group is nearly coplanar with the heterocyele ring with the dihedral angle of 4°. The N2 atom is protonated with N2···H distance of 0.89 (3) Å. The ions are linked into one chain through intermolecular hydrogen bonds [O1—H1···Cl1^i^ and N2—H2A···Cl1^ii^; symmetry code: i = 2 - *x*,1 - *y*,1 - *z*; ii = -*x* + 1, *y* + 1/2, -*z* + 3/2.] (shown in Fig. 2). The crystal structure is stabilized by van der waals forces (shown in Fig. 3).

### Experimental

To a ethanol solution of 2-aminopyridine (7.21 g, 0.0766 mol) under nitrogen was added ethyl 4-chloroacetoacetate (6 g, 0.0365 mol). The mixture was refluxed for 2 h before concentrated to dryness. The residue was dissolved in 80 ml of purified water and extracted with ethyl acetate. The organic phase was concentrated to give a black oily consistency. 30% KOH (153 ml) was added and stirred for 3 h at 40 *^o^*C. The crystals will form after adding concentrated HCl.

### Refinement

Carbon-bond H atoms were positioned geometrically (C—H = 0.93 Å for phenyl group, C—H = 0.93 Å for imidazole group), and were included in the refinement in the riding mode approximation, with *U*_iso_(H) = 1.2*U*_eq_(C) for imidazole group and phenyl group. H atoms bound to O and N atoms were located in a difference Fourier map.

### Crystal data

**Table:**

C_9_H_9_N_2_O_2_^+^·Cl^−^	*F*(000) = 440
*M**_r_* = 212.63	*D*_x_ = 1.475 Mg m^−^^3^*D*_m_ = 1.475 Mg m^−^^3^*D*_m_ measured by not measured
Monoclinic, *P*2_1_/*c*	Mo *K*α radiation, λ = 0.71073 Å
Hall symbol: -P 2ybc	Cell parameters from 9784 reflections
*a* = 5.4032 (8) Å	θ = 3.2–25.0°
*b* = 14.722 (2) Å	µ = 0.37 mm^−^^1^
*c* = 12.1055 (18) Å	*T* = 293 K
β = 96.182 (4)°	Block, yellow
*V* = 957.3 (2) Å^3^	0.25 × 0.15 × 0.12 mm
*Z* = 4	

### Data collection

**Table:**

Rigaku Mercury diffractometer	1689 independent reflections
Radiation source: fine-focus sealed tube	1417 reflections with *I* > 2σ(*I*)
Graphite monochromator	*R*_int_ = 0.044
/w scans	θ_max_ = 25.0°, θ_min_ = 3.2°
Absorption correction: multi-scan (*CrystalClear*; Rigaku, 2005)	*h* = −6→6
*T*_min_ = 0.913, *T*_max_ = 0.957	*k* = −17→17
7948 measured reflections	*l* = −14→14

### Refinement

**Table:**

Refinement on *F*^2^	Primary atom site location: structure-invariant direct methods
Least-squares matrix: full	Secondary atom site location: difference Fourier map
*R*[*F*^2^ > 2σ(*F*^2^)] = 0.047	Hydrogen site location: inferred from neighbouring sites
*wR*(*F*^2^) = 0.083	H atoms treated by a mixture of independent and constrained refinement
*S* = 1.02	*w* = 1/[σ^2^(*F*_o_^2^) + (0.0102*P*)^2^ + 1.0216*P*] where *P* = (*F*_o_^2^ + 2*F*_c_^2^)/3
1689 reflections	(Δ/σ)_max_ < 0.001
134 parameters	Δρ_max_ = 0.19 e Å^−^^3^
0 restraints	Δρ_min_ = −0.20 e Å^−^^3^

### Special details

**Table:**

Geometry. All e.s.d.'s (except the e.s.d. in the dihedral angle between two l.s. planes) are estimated using the full covariance matrix. The cell e.s.d.'s are taken into account individually in the estimation of e.s.d.'s in distances, angles and torsion angles; correlations between e.s.d.'s in cell parameters are only used when they are defined by crystal symmetry. An approximate (isotropic) treatment of cell e.s.d.'s is used for estimating e.s.d.'s involving l.s. planes.
Refinement. Refinement of *F*^2^ against ALL reflections. The weighted *R*-factor *wR* and goodness of fit *S* are based on *F*^2^, conventional *R*-factors *R* are based on *F*, with *F* set to zero for negative *F*^2^. The threshold expression of *F*^2^ > σ(*F*^2^) is used only for calculating *R*-factors(gt) *etc*. and is not relevant to the choice of reflections for refinement. *R*-factors based on *F*^2^ are statistically about twice as large as those based on *F*, and *R*- factors based on ALL data will be even larger.

### Fractional atomic coordinates and isotropic or equivalent isotropic displacement parameters (Å^2^)

**Table:**

	*x*	*y*	*z*	*U*_iso_*/*U*_eq_	
Cl1	0.99075 (13)	0.33311 (5)	0.93211 (6)	0.0507 (2)	
C1	0.3725 (4)	0.61391 (17)	0.6832 (2)	0.0372 (6)	
C6	0.6807 (5)	0.57805 (17)	0.5799 (2)	0.0396 (6)	
H6	0.8196	0.5503	0.5556	0.048*	
O1	0.7448 (4)	0.72008 (14)	0.26206 (16)	0.0559 (6)	
H1	0.8352	0.6986	0.2184	0.084*	
O2	0.9053 (3)	0.60844 (14)	0.37360 (15)	0.0540 (5)	
N1	0.5729 (4)	0.55760 (14)	0.67658 (17)	0.0363 (5)	
C5	0.6379 (5)	0.49364 (18)	0.7568 (2)	0.0431 (7)	
H2	0.7747	0.4561	0.7521	0.052*	
C4	0.4994 (5)	0.48630 (19)	0.8428 (2)	0.0482 (7)	
H3	0.5406	0.4428	0.8974	0.058*	
C3	0.2926 (5)	0.5438 (2)	0.8513 (2)	0.0496 (7)	
H4	0.1992	0.5377	0.9110	0.060*	
C2	0.2295 (5)	0.60797 (19)	0.7725 (2)	0.0451 (7)	
H5	0.0954	0.6467	0.7779	0.054*	
N2	0.3549 (4)	0.66686 (16)	0.59268 (18)	0.0402 (5)	
C7	0.5447 (5)	0.64601 (17)	0.5281 (2)	0.0374 (6)	
C8	0.5594 (5)	0.69786 (18)	0.4237 (2)	0.0451 (7)	
H8A	0.5862	0.7613	0.4428	0.054*	
H8B	0.3994	0.6935	0.3792	0.054*	
C9	0.7576 (5)	0.66897 (19)	0.3528 (2)	0.0419 (6)	
H2A	0.251 (6)	0.714 (2)	0.581 (2)	0.067 (10)*	

### Atomic displacement parameters (Å^2^)

**Table:**

	*U*^11^	*U*^22^	*U*^33^	*U*^12^	*U*^13^	*U*^23^
Cl1	0.0547 (4)	0.0434 (4)	0.0564 (4)	−0.0043 (3)	0.0173 (3)	0.0008 (3)
C1	0.0329 (14)	0.0375 (15)	0.0410 (15)	−0.0032 (11)	0.0026 (12)	−0.0069 (12)
C6	0.0347 (14)	0.0426 (16)	0.0429 (15)	0.0031 (12)	0.0099 (12)	−0.0034 (12)
O1	0.0640 (14)	0.0566 (13)	0.0500 (12)	0.0101 (10)	0.0193 (10)	0.0099 (11)
O2	0.0505 (12)	0.0654 (14)	0.0475 (11)	0.0186 (11)	0.0110 (10)	0.0049 (10)
N1	0.0337 (11)	0.0358 (12)	0.0393 (12)	−0.0016 (10)	0.0035 (10)	−0.0030 (10)
C5	0.0395 (15)	0.0441 (16)	0.0448 (16)	0.0022 (12)	0.0002 (13)	0.0016 (13)
C4	0.0530 (18)	0.0477 (17)	0.0430 (16)	−0.0026 (14)	0.0010 (14)	0.0028 (13)
C3	0.0514 (17)	0.0566 (19)	0.0422 (16)	−0.0097 (15)	0.0113 (14)	−0.0047 (14)
C2	0.0400 (15)	0.0484 (17)	0.0484 (16)	0.0007 (13)	0.0115 (13)	−0.0100 (14)
N2	0.0363 (12)	0.0401 (13)	0.0446 (13)	0.0055 (11)	0.0066 (10)	−0.0019 (11)
C7	0.0346 (13)	0.0371 (15)	0.0408 (14)	−0.0023 (11)	0.0064 (12)	−0.0061 (12)
C8	0.0454 (16)	0.0442 (16)	0.0459 (16)	0.0053 (13)	0.0064 (13)	0.0007 (13)
C9	0.0410 (15)	0.0432 (16)	0.0412 (15)	−0.0040 (13)	0.0033 (12)	−0.0036 (13)

### Geometric parameters (Å, º)

**Table:**

C1—N2	1.340 (3)	C4—C3	1.414 (4)
C1—N1	1.373 (3)	C4—H3	0.9300
C1—C2	1.398 (4)	C3—C2	1.359 (4)
C6—C7	1.354 (3)	C3—H4	0.9300
C6—N1	1.395 (3)	C2—H5	0.9300
C6—H6	0.9300	N2—C7	1.389 (3)
O1—C9	1.327 (3)	N2—H2A	0.89 (3)
O1—H1	0.8200	C7—C8	1.487 (4)
O2—C9	1.205 (3)	C8—C9	1.503 (4)
N1—C5	1.371 (3)	C8—H8A	0.9700
C5—C4	1.351 (4)	C8—H8B	0.9700
C5—H2	0.9300		
			
N2—C1—N1	106.9 (2)	C3—C2—C1	117.9 (3)
N2—C1—C2	132.3 (2)	C3—C2—H5	121.0
N1—C1—C2	120.8 (2)	C1—C2—H5	121.0
C7—C6—N1	107.0 (2)	C1—N2—C7	109.9 (2)
C7—C6—H6	126.5	C1—N2—H2A	124 (2)
N1—C6—H6	126.5	C7—N2—H2A	125 (2)
C9—O1—H1	109.5	C6—C7—N2	107.4 (2)
C5—N1—C1	121.1 (2)	C6—C7—C8	134.2 (2)
C5—N1—C6	130.1 (2)	N2—C7—C8	118.4 (2)
C1—N1—C6	108.8 (2)	C7—C8—C9	116.5 (2)
C4—C5—N1	118.7 (3)	C7—C8—H8A	108.2
C4—C5—H2	120.7	C9—C8—H8A	108.2
N1—C5—H2	120.7	C7—C8—H8B	108.2
C5—C4—C3	121.0 (3)	C9—C8—H8B	108.2
C5—C4—H3	119.5	H8A—C8—H8B	107.3
C3—C4—H3	119.5	O2—C9—O1	124.5 (2)
C2—C3—C4	120.4 (3)	O2—C9—C8	125.9 (3)
C2—C3—H4	119.8	O1—C9—C8	109.6 (2)
C4—C3—H4	119.8		

### Hydrogen-bond geometry (Å, º)

**Table:**

*D*—H···*A*	*D*—H	H···*A*	*D*···*A*	*D*—H···*A*
O1—H1···Cl1^i^	0.82	2.19	2.984 (2)	163
N2—H2*A*···Cl1^ii^	0.89 (3)	2.18 (3)	3.074 (2)	175 (3)

Symmetry codes: (i) −*x*+2, −*y*+1, −*z*+1; (ii) −*x*+1, *y*+1/2, −*z*+3/2.
